# Prevalence and features of hypervirulent *Klebsiella pneumoniae* in respiratory specimens at a US hospital system

**DOI:** 10.1128/iai.00486-24

**Published:** 2024-12-11

**Authors:** Christi L. McElheny, Alina Iovleva, Nathalie Chen, Dominic Woods, Akansha Pradhan, Jonah L. Sonnabend, Aidan R. Matunis, Nathan J. Raabe, Janet S. Lee, Giraldina Trevejo-Nuñez, Daria Van Tyne, Yohei Doi

**Affiliations:** 1 Center for Innovative Antimicrobial Therapy, Division of Infectious Diseases, University of Pittsburgh School of Medicine 12317 , Pittsburgh, Pennsylvania, USA; 2 Division of Pulmonary and Critical Care Medicine, Washington University School of Medicine 677234 , St. Louis, Missouri, USA; 3 Center for Evolutionary Biology and Medicine, University of Pittsburgh School of Medicine 607640 , Pittsburgh, Pennsylvania, USA; 4 Departments of Microbiology, Fujita Health University School of Medicine 12695 , Toyoake, Aichi, Japan; 5 Departments of Infectious Diseases, Fujita Health University School of Medicine 89305 , Toyoake, Aichi, Japan; University of California Davis, Davis, California, USA

**Keywords:** hypervirulence, *Klebsiella*, surveillance

## Abstract

Hypervirulent *Klebsiella pneumoniae* (hvKp) strains are considered to be relatively rare in the United States, but cases are increasingly reported. We prospectively and serially collected *K. pneumoniae* clinical isolates identified in respiratory specimens at a health system in Western Pennsylvania between 2020 and 2022. A total of 273 *K*. *pneumoniae* isolates from 216 unique patients were analyzed for markers of hypervirulence by both string test for a hypermucoid phenotype and multiplex PCR to detect isolates carrying cardinal virulence genes *rmpA*, *rmpA2*, *iutA*, and *iro*. Of the 273 isolates, 13 (4.8%) tested positive by string test including 11 nonduplicate *K. pneumoniae* isolates, and two of these (0.7%) were positive by PCR for virulence genes *rmpA*, *rmpA2*, *iutA*, and *iro*. The latter two putative hvKp strains, belonging to sequence types ST23-K1 and ST86-SLV-K2, possessed pLVPK-like plasmids, and were collected from community-associated infections in individuals without known travel histories. Both putative hvKp strains and two additional string test-positive strains were resistant to killing by human serum. The hvKp strains caused significant pneumonia in mice infected by oropharyngeal aspiration, with significantly higher weight loss and increased bacterial burden in the lungs of mice infected with the KL1 (ST23) strain compared to the KL2 (ST86-SLV) strain. We also observed decreased survival of mice infected with the KL1 strain compared to the KL2 strain. These findings add to the growing body of evidence suggesting that hvKp strains, once considered endemic to Asia, may now be circulating in North America.

**IMPORTANCE:**

Certain lineages of *Klebsiella pneumoniae* are increasingly recognized to cause severe community-associated infection, but information on their prevalence in the United States is limited. In a prospective, sequential cohort of 273 *K*. *pneumoniae* respiratory isolates, we identified two of them as genetically defined hypervirulent *K. pneumoniae*. The isolates were from local residents who developed community-onset pneumonia, suggesting that hypervirulent *K. pneumoniae* may already be present in the community.

## INTRODUCTION

*Klebsiella pneumoniae* is a major cause of healthcare-associated infections including nosocomial pneumonia, bacteremia, and urinary tract infection (1). *K. pneumoniae* has increasingly become multidrug-resistant and is now considered one of the most problematic gram-negative bacteria in the healthcare environment. On the other hand, *K. pneumoniae* has also been recognized as a community-associated pathogen responsible for invasive infections such as severe community-acquired pneumonia, pyogenic liver abscess, and endogenous endophthalmitis, first in East Asia then in other parts of the world as well (1). These community-associated infections are typically caused by hypervirulent strains belonging to distinct lineages, in particular, those possessing capsular serotypes K1 and K2 and corresponding to sequence types (ST) 23 and ST65/66/86, respectively. Hypervirulent *K. pneumoniae* (hvKp) strains often carry genes associated with aerobactin siderophore biosynthesis (*iuc*), salmochelin biosynthesis (*iro*), metabolic transporter (*peg-344*), and regulators of the mucoid phenotype (*rmpA/rmpA2*) (2).

hvKp strains are considered to be relatively rare in the United States, but cases are increasingly reported (3, 4). Systematic sequencing of 104 unique *K. pneumoniae* bloodstream infection isolates in a Chicago area hospital collected between 2015 and 2017 identified four hvKp strains, belonging to ST23, ST66, and ST380 (5). Two and one of these patients had abscesses in the liver and the lung, respectively. Another longitudinal survey of 463 isolates, mostly originating from blood and collected between 2015 and 2018 at a hospital in New York City, reported 16 isolates with capsular serotypes associated with hvKp (6). In all of these studies, the hvKp strains were also hypermucoviscous, but not all hypermucoviscous strains were hvKp, as previously reported (2).

While the spread of hvKp is a potential public health concern, studies to date in the United States have mostly surveyed archived blood isolates. While *K. pneumoniae* is a major cause of pneumonia in both community and healthcare settings, the prevalence of hvKp and its trajectory among respiratory tract infections remain unclear. We therefore screened sequential *K. pneumoniae* clinical strains isolated from respiratory specimens at a university hospital system in Pennsylvania between 2020 and 2022 to estimate the prevalence of hvKp and investigate their genomic and phenotypic features.

## MATERIALS AND METHODS

### Isolate selection

A total of 273 *K*. *pneumoniae* isolates from 216 unique patients that were cultured from respiratory specimens and available (sputum, bronchoalveolar lavage, and tracheal aspirate) were serially collected between May 2020 and June 2022 from the central clinical microbiology laboratory of the University of Pittsburgh Medical Center, a university hospital system located in Western Pennsylvania. The study was approved by the Institutional Review Board of the University of Pittsburgh (STUDY20030071 and STUDY20110023).

### String test

Bacterial isolates were cultured on blood agar plates and incubated overnight at 37°C. An inoculating loop was used to gently touch and lift individual colonies. A positive test result was defined as a mucoid string >5 mm in length that was observed visually (7).

### Multiplex PCR

Multiplex PCR was performed on all isolates using previously designed primers and reaction conditions to detect *rmpA*, *rmpA2*, *iroN*, and *iutA*, which are cardinal virulence genes frequently located on the pLVPK virulence plasmid (1, 8). The IncHI1B replicon gene, which is also associated with pLVPK, was included in the multiplex reaction as well.

### Antimicrobial susceptibility testing

Antimicrobial susceptibility testing was performed by the disk diffusion method using Sensi-Disc disks (BD, Franklin Lakes, NJ, USA) at least in duplicate. Results were interpreted according to Clinical and Laboratory Standards Institute (CLSI) breakpoints (9), except for tigecycline which was interpreted according to the Food and Drug Administration (FDA) breakpoints.

### Whole genome sequencing and analysis

**Fig 1:**
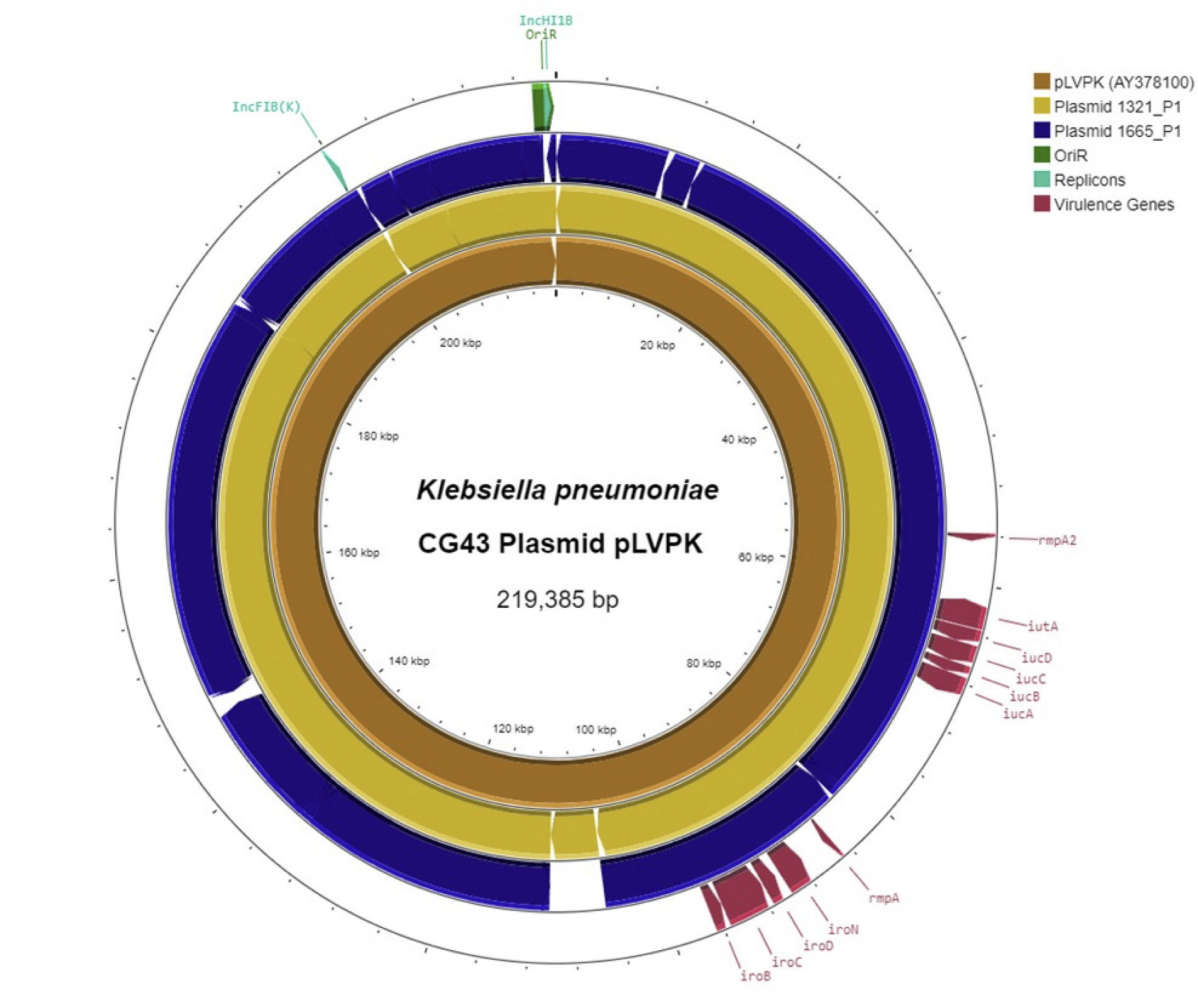
Study plasmids aligned to the reference pLVPK plasmid. An alignment between study plasmids 1321_P1 and 1665_P1 (middle and outer circles) to the reference plasmid pLVPK (inner circle) was generated using BLASTn and visualized using Proksee. Conserved virulence genes (visualized in red) and plasmid replicons (visualized in green) were annotated using Bakta, PlasmidFinder, and BLASTn.

String test-positive isolates were subjected to whole genome sequencing on a NextSeq500 (Illumina, San Diego, CA, USA). Sequencing libraries (150 bp paired end) were prepared as described with minor modifications (10). Additionally, two isolates (1321 and 1665) were sequenced with long-read technology on an Oxford Nanopore MinION device (Oxford Nanopore Technologies, Oxford, United Kingdom). Hybrid assembly was conducted for the genomes of these two isolates using unicycler v0.4.8-beta (11). Hybrid assembled genomes were annotated using RAST (12). Kleborate was used to identify STs, resistance genes, and virulence factors (13). For plasmid sequence comparison, an annotated alignment of study plasmids 1321_P1 and 1665_P1 to the NCBI reference pLVPK plasmid (AY378100) was created and visualized using Proksee (Fig. 1) (14). Plasmid replicons were assigned via BLASTn hits of ≥80% similarity to identifiable replicons in the PlasmidFinder database, and annotation of virulence genes was accomplished using BLASTn and Bakta (15). Plasmid relatedness was evaluated using BLASTn unidirectional ([identity] × [coverage]/100) and normalized bidirectional ([similarity plasmid A:B] × [similarity plasmid B:A]/100) similarity scores.

### Serum susceptibility

Overnight cultures of bacterial isolates were diluted 1:100 in lysogenic broth (LB) and grown at 37°C to the optical density at 600 nm (OD_600_) of at least 0.2. Bacteria were pelleted by centrifugation and resuspended in 1× sterile phosphate buffered saline (PBS) to OD_600_ of 0.2. Four microliters of bacteria was combined with 2 µL of LB and 34 µL of normal human serum (NHS) (Complement Technology, Tyler, TX, USA). The negative control condition (no NHS) used 34 µL of 1× PBS in place of serum. Samples were incubated at 37°C for 3 hours. After incubation, samples were serially diluted in PBS and plated on LB agar to obtain colony counts. Isolate susceptibility to NHS was categorized as “strongly susceptible,” “moderately susceptible,” or “resistant.” Resistant isolates had similar or greater concentrations after incubation in NHS compared to the no NHS condition. Strongly susceptible isolates had concentrations within 1,000-fold of the limit of detection. Moderately susceptible isolates had concentrations lower than the no NHS condition but were not as extensively killed as the strongly susceptible isolates. Each isolate was tested in two biological replicates, each with three technical replicates.

### Mouse studies

Ten-week-old C57BL/6J female mice were purchased from the Jackson Labs and used in experiments. Briefly, *K. pneumoniae* strains of interest from this cohort (1321, 1665, 2127, and 790) and a control (non-mucoid, KPC-producing ST258 strain C4 (16)) were grown overnight in LB and then subcultured for 2 hours at 37°C with shaking to reach an OD_600_ of 0.5. Bacteria were washed three times and resuspended in PBS. Mice were anesthetized with isoflurane, and 1–2 × 10^3^ colony forming units (CFUs) of bacteria suspended in 50 µL of PBS was administered by deep oropharyngeal aspiration. Mice were sacrificed at 48 hours post-infection. The lungs, liver, and spleen of each mouse were collected, homogenized, and plated on LB agar by serial dilution to determine CFUs per organ. Data were analyzed on Prism (GraphPad) by one-way analysis of variance (ANOVA) with multiple comparison tests.

## RESULTS

### Overview

**Fig 2:**
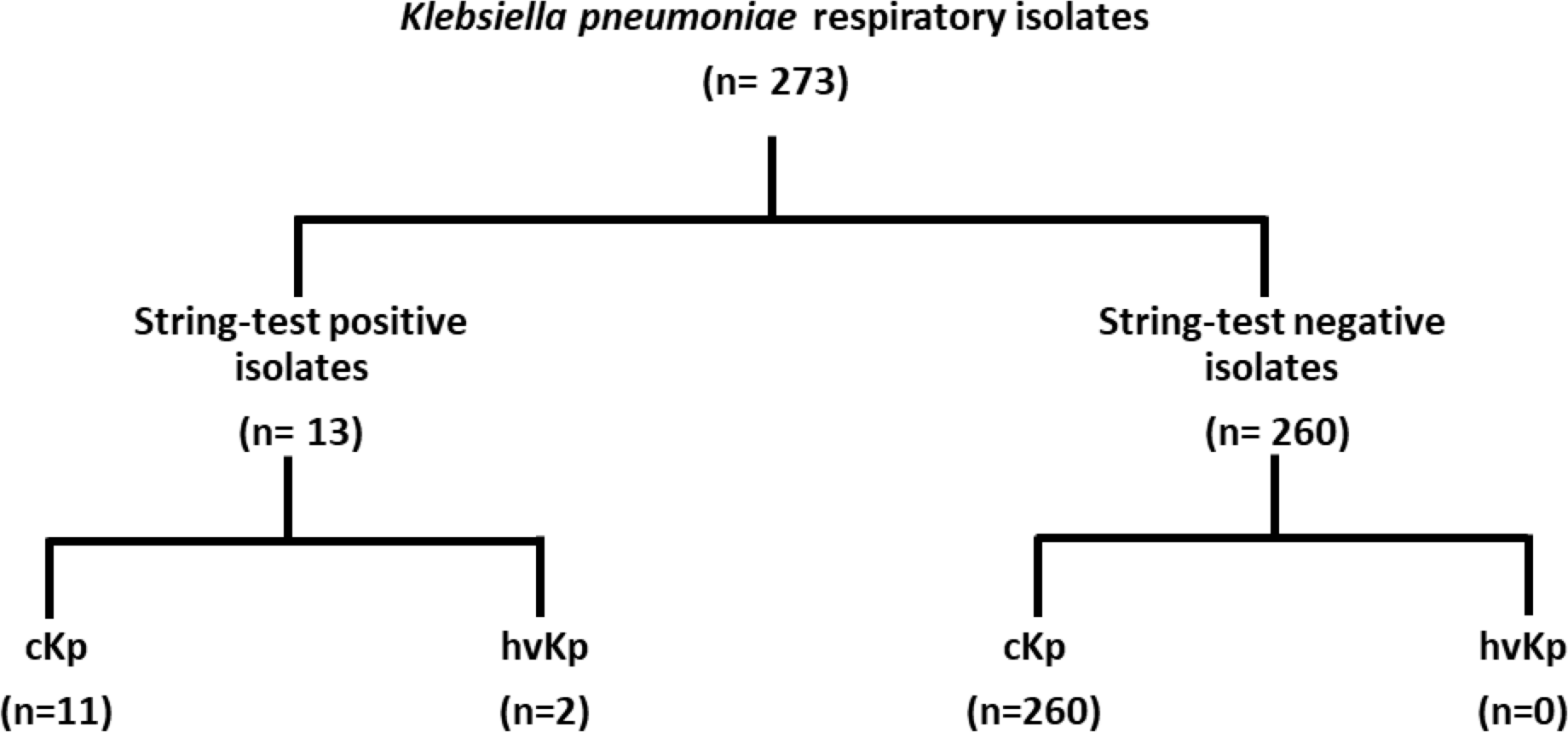
Screening of *Klebsiella pneumoniae* clinical isolates by string test and multiplex PCR. cKp, classical *K. pneumoniae*; hvKp, hypervirulent *K. pneumoniae*.

A total of 273 *K*. *pneumoniae* isolates from 216 unique patients were analyzed by both string test for hypermucoidal phenotype and by multiplex PCR to detect isolates carrying virulence genes *rmpA*, *rmpA2*, *iutA*, and *iroN*. Of the 273 isolates, 13 (4.8%) tested positive by string test, including one strain which was reclassified as *Klebsiella quasipneumoniae* upon whole genome sequencing (1700) and two strains that were collected from the same patient (2127 and 2148) and assumed to be duplicates of one another. Thus, there were 11 unique string test-positive *K. pneumoniae* isolates, two (0.7%) of which were positive by PCR for the four cardinal virulence genes (Fig. 2). These two strains (1321 and 1665) carried *rmpA*, truncated *rmpA2*, *iutA*, and *iroN*. For the remaining string test-positive isolates and the 260 isolates that were negative by string test, none of the four cardinal virulence genes were detected by PCR.

### Antimicrobial susceptibility

**Fig 3:**
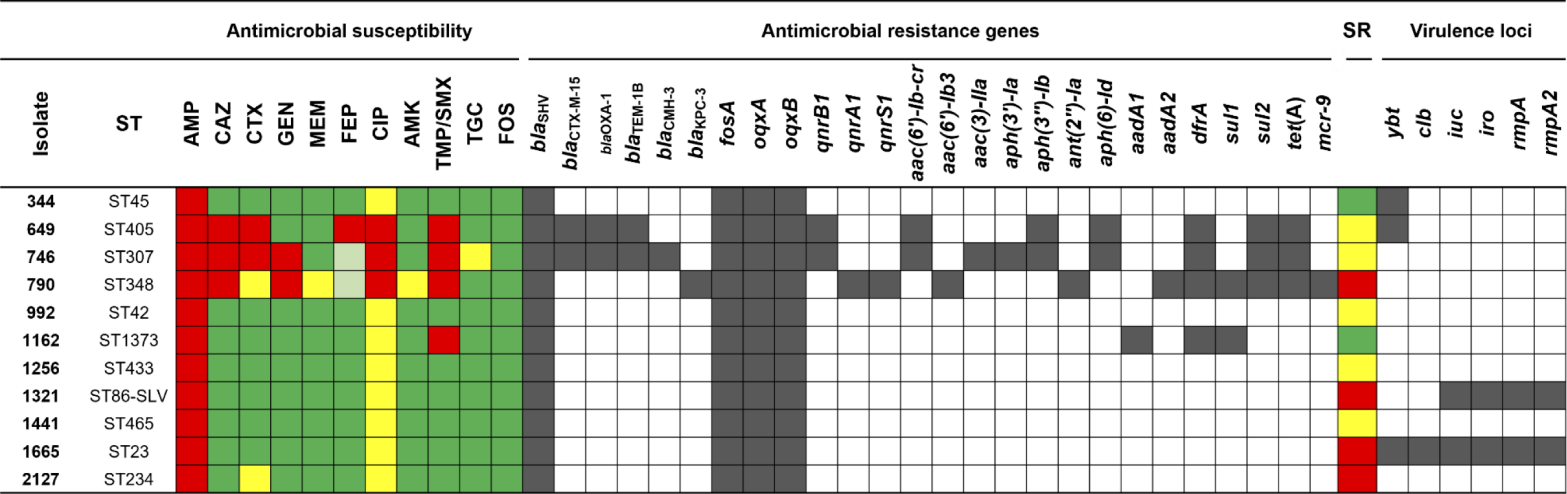
ST, antimicrobial susceptibility, antimicrobial resistance genes, serum resistance, and virulence genes identified in hypermucoviscous *Klebsiella pneumoniae* isolate genomes. Green, susceptible; light green, susceptible dose-dependent; yellow, intermediate; red, resistant for antimicrobial susceptibility and serum resistance (SR). AMP, ampicillin; CAZ, ceftazidime; CTX, cefotaxime; GEN, gentamicin; MEM, meropenem; FEP, cefepime; CIP, ciprofloxacin; AMK, amikacin; TGC, tigecycline; FOS, fosfomycin. Susceptibility was interpreted according to the CLSI breakpoints, except for tigecycline, which was interpreted according to the FDA.

Of the 11 string test-positive strains, one strain (790) showed intermediate susceptibility to meropenem, and this and two additional strains (649 and 746) were resistant to ceftazidime and ciprofloxacin (Fig. 3). The latter two strains met the criteria for extended-spectrum β-lactamase (ESBL) production. These three strains were multidrug-resistant, while the remaining eight strains were susceptible to all agents tested except for ciprofloxacin, which was interpreted as intermediate based on recently updated CLSI breakpoints (9).

### Molecular epidemiology and resistance/virulence genes

We performed whole genome sequencing on the 11-string test-positive strains. All strains except two belonged to different multi-locus STs and had different *wzi* loci and K loci (Table 1). Strains 746 (ST307) and 1256 (ST433, a double locus variant of ST307) both encoded *wzi*173 and KL102. The carbapenem-non-susceptible strain 790 belonged to ST348 and possessed *bla*_KPC-3_, a combination that has been reported in a hospital outbreak in Portugal (17). The two ESBL-producing isolates carried *bla*_CTX-M-15_ and belonged to ST307 and ST405, both well-recognized multidrug-resistant lineages (18, 19). All trimethoprim-sulfamethoxazole-resistant isolates carried a combination of *sul* and *dfr* genes. Interestingly, *mcr-9* was also carried by the *bla*_KPC-3_-positive isolate.

**TABLE 1:** Serum susceptibility testing and virulence-associated features of string test-positive strains^*a*^

Strain	ST	Serum susceptibility	Virulence score	Acquired virulence loci	Capsule			hvKp plasmids	Clinical condition
					*wzi*	K locus	O locus		
344	ST45	Strongly susceptible	1	*ybt*	*wzi*101	KL24	O1/O2v1	–^*c*^	Aspiration pneumonia
649	ST405	Moderately susceptible	1	*ybt*	*wzi*424	KL151	O4	–	Polymicrobial hospital-acquired pneumonia
746	ST307	Moderately susceptible	0	–	*wzi*173	KL102	O1/O2v2	–	Routine surveillance bronchoscopy on transplant patient
790	ST348	Resistant	0	–	*wzi*94	KL62	O1/O2v1	–	Recurrent healthcare-associated pneumonia
992	ST42	Moderately susceptible	0	–	*wzi*98	KL122	O1/O2v2	–	Polymicrobial ventilator-associated pneumonia
1162	ST1373	Strongly susceptible	0	–	*wzi*96	KL38	O3b	–	Routine surveillance bronchoscopy on transplant patient
1256	ST433	Moderately susceptible	0	–	*wzi*173	KL102	O1/O2v2	–	Aspiration pneumonia
1321	ST86- SLV	Resistant	3	*iuc*, *iro*, *rmpA rmpA2* (truncated)	*wzi*2	KL2^*b*^	O1/O2v1	pLVPK-like	Aspiration pneumonia
1441	ST465	Moderately susceptible	0	–	*wzi*115	KL54	O1/O2v2	–	Polymicrobial hospital-acquired pneumonia
1665	ST23	Resistant	5	*ybt*, *clb*, *iuc*, *iro*, *rmpA*, *rmpA2* (truncated)	*wzi*1	KL1	O1/O2v2	pLVPK-like	Aspiration pneumonia
2127	ST234	Resistant	0	–	*wzi*114	KL30^*b*^	O1/O2v2	–	Recurrent hospital-acquired pneumonia

^*a*^ *ybt*, yerseniabactin gene loci; *iro*, salmochelin biosynthesis gene loci; *iuc*, aerobactin synthesis gene loci; *clb*, colibactin synthesis gene loci; *rmpA2*, regulator of mucoid phenotype gene loci.

^*b*^ Kleborate indicated imperfect match.

^*c*^ "–" indicates empty cells.

Of the two isolates carrying the cardinal virulence-associated genes, strain 1321 was a single locus variant of ST86 and encoded the KL2 capsular locus. The other strain, 1665, belonged to ST23 and encoded the KL1 capsule locus. The two putative hvKp strains carried intrinsic resistance genes *bla*_SHV_, *oqxA/B*, and *fosA*, and were only resistant to ampicillin (Fig. 3). In both strains, the virulence-associated genes were located on pLVPK-like plasmids (1665_P1 and 1321_P1). When compared to the reference pLVPK plasmid AY378100 (219,385 bp), the shorter study plasmid 1665_P1 (223,655 bp) was more similar (bidirectional similarity = 86.8%) than the longer plasmid 1321_P1 (226,989 bp) (bidirectional similarity = 47.9%). Both study plasmids contained unique additional regions and rearrangements compared to the reference pLVPK plasmid; however, unidirectional comparisons showed that a large proportion of the reference pLVPK plasmid backbone was represented in both study plasmids (82.5% similarity with pLVPK:1321_P1 and 94.1% similarity with pLVPK:1665_P1), which included shared IncFIB(K)/IncHI1B replicons and virulence genes *iroBCDN*, *repA/repA2*, *iucABCD*, and *iutA*. When the two study plasmids were compared directly to one another, 1321_P1 and 1665_P1 had high unidirectional similarity scores of 90.9% (1321_P1:1665_P1) and 92.2% (1665_P1:1321_P1), respectively, resulting in a normalized bidirectional similarity score of 83.9%.

### Serum resistance

**Fig 4:**
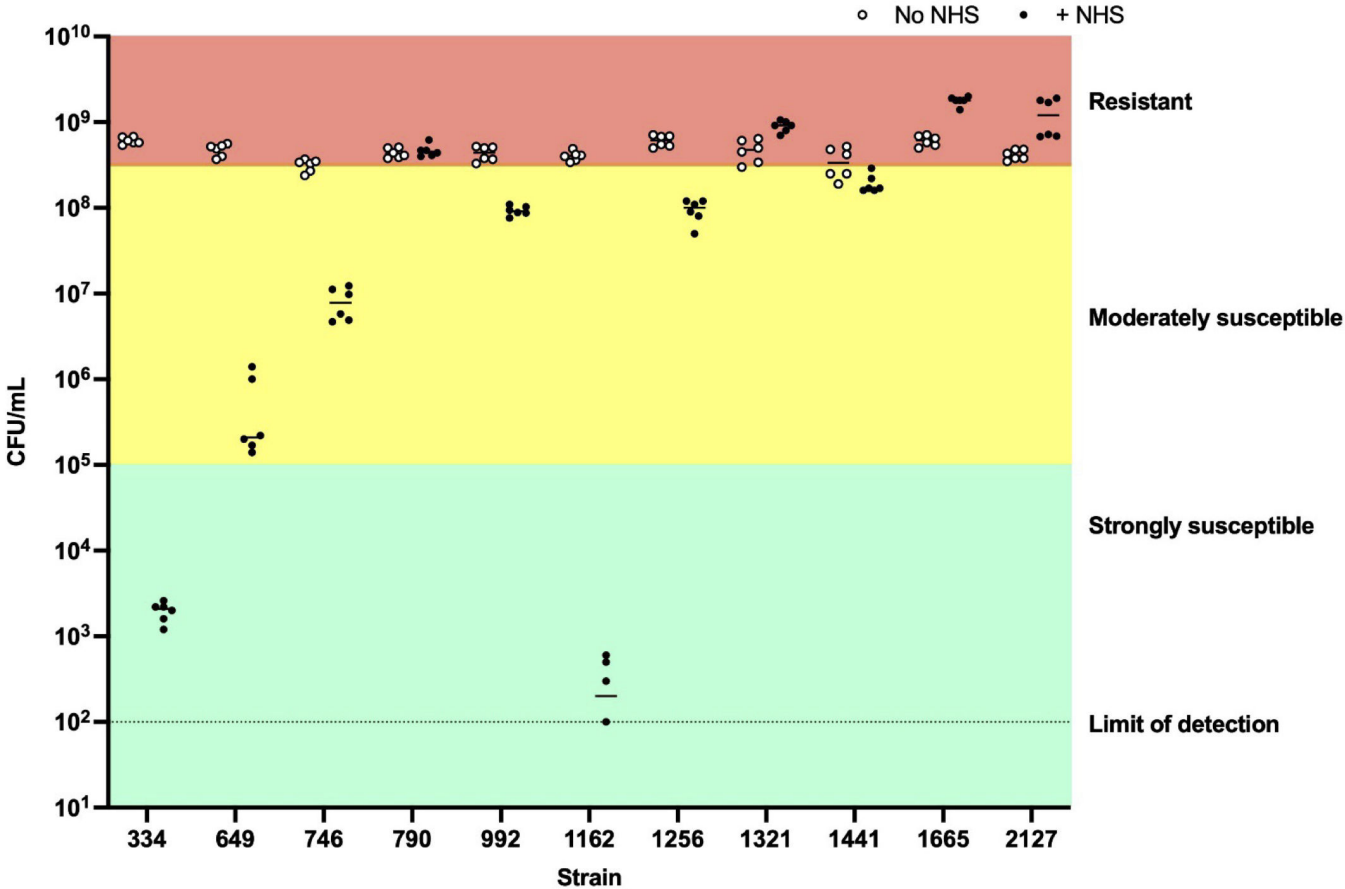
Human serum susceptibility of string test-positive isolates. Isolate susceptibility to NHS was categorized as “strongly susceptible,” “moderately susceptible,” or “resistant.” Resistant isolates had similar or greater viability after incubation in NHS compared to the no NHS condition. Strongly susceptible isolates had concentrations within 1,000-fold of the limit of detection. Moderately susceptible isolates had concentrations lower than the no NHS condition but were not as extensively killed as the strongly susceptible isolates. Each isolate was tested in two biological replicates, each with three technical replicates.

To evaluate whether a string test-positive phenotype correlated with resistance to killing by serum, all string test-positive strains, along with previously established serum-sensitive and serum-resistant control strains (data not shown), were tested for their *in vitro* susceptibility to NHS. Four of 11 strains were serum-resistant, including the genetically defined hvKp strains 1321 and 1665 (Fig. 4). The remaining serum-resistant strains included the carbapenem-resistant strain 790 and strain 2127, neither of which carried any acquired virulence genes (Fig. 3). Five strains were moderately susceptible to serum, and the remaining three strains were highly susceptible to serum.

### Virulence of string test-positive, serum-resistant *K. pneumoniae* strains

**Fig 5:**
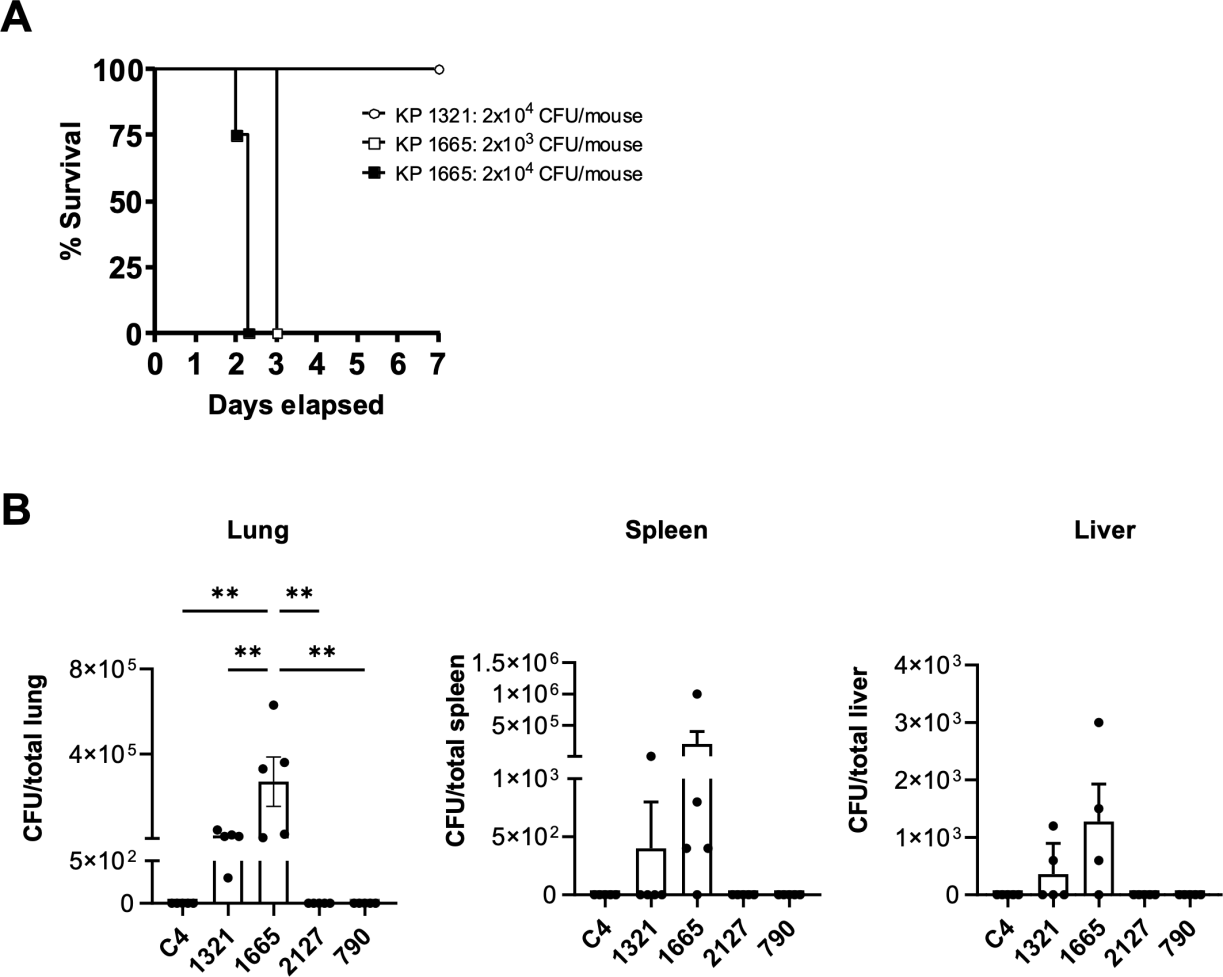
Virulence of *K. pneumoniae* strains in a mouse pneumonia model. (**A**) Mice were infected with 2 × 10^3^ to 2 × 10^4^ CFUs of either the KL1 (1665) or KL2 (1321) strain, and survival was followed for 7 days. (**B**) Mice were infected with 1–2 × 10^3^ CFUs of different strains of *K. pneumoniae*, including the non-mucoid, serum-sensitive ST258 strain C4, putative hvKp strains 1321 and 1665, and non-hvKp, string test-positive, serum-resistant strains 2127 and 790. Mice were sacrificed at 48 hours post-infection, and whole organs were processed for CFU plating. Each dot represents one mouse. **P < 0.01, one-way ANOVA.

We compared the virulence of our KL1 (1665) and KL2 (1321) genetically defined hvKp strains *in vivo*. We infected wild-type mice with 2 × 10^3^ or 2 × 10^4^ CFUs of bacteria and followed their survival for 7 days. Inoculation of 2 × 10^3^ or 2 × 10^4^ CFUs of the KL1 (1665) strain resulted in 25% and 100% mortality, respectively, at 2 days, with 100% mortality observed at day 3 for both groups. However, all mice infected with the higher dose of 2 × 10^4^ CFUs of the KL2 (1321) strain survived until day 7 (Fig. 5A). We also investigated if the string test-positive, serum-resistant *K. pneumoniae* strains not genetically defined as hvKp behaved as hvKp strains *in vivo*. Wild-type mice were infected with a non-mucoid ST258 strain (C4), the serum-resistant, genetically defined hvKp strains 1331 and 1665, and the serum-resistant, string test-positive *K. pneumoniae* strains 790 and 2127. The five mice in each group were each infected with 1–2 × 10^3^ CFUs of bacteria and were sacrificed at 48 hours post-infection. As shown, strain 1665 had the highest bacterial burden in lungs, followed by strain 1321. On the other hand, *K. pneumoniae* strains 790 and 2127 displayed no tissue growth above the limit of detection at 48 hours post-infection (Fig. 5B).

### Cases associated with genetically defined hypervirulent strains

Both of the putative hvKp cases occurred in 2021. The patient from whom strain 1321 (ST86-SLV, KL2) was collected was an adult who was found pulseless at home, resuscitated on the scene, and intubated upon arrival at the hospital. The chest radiograph was consistent with aspiration pneumonia, for which piperacillin-tazobactam and vancomycin were started. Due to continued fever and increasing oxygen requirements, a sputum culture was collected on hospital day 4, which grew *K. pneumoniae* and *Stenotrophomonas maltophilia*. The treatment regimen was changed to ampicillin-sulbactam, the patient’s respiratory and neurologic statuses improved, and the patient was eventually discharged to a rehabilitation facility.

The patient from whom strain 1665 (ST23, KL1) was collected was also an adult who was found pulseless at home and was resuscitated and intubated upon arrival at the hospital. The chest radiograph was consistent with aspiration pneumonia. The patient had suffered an anoxic brain injury and was pronounced dead on hospital day 3; however, a bronchoalveolar lavage culture collected on that day grew *K. pneumoniae*. Both patients presented from the local community and had histories of recreational drug use, which was presumed to be the cause of their cardiac events. Neither patient had any documented history of overseas travel, suggesting local community acquisition of their infecting *K. pneumoniae* strains.

## DISCUSSION

While *K. pneumoniae* has primarily been considered as a healthcare-associated and antibiotic-resistant pathogen, hvKp has also drawn attention as a cause of invasive community-associated infections including pneumonia, liver abscess, and endophthalmitis (1). hvKp infections are reported mostly from Asia and are often associated with specific lineages of *K. pneumoniae* like ST23, ST65, and ST86. hvKp also often carries a plasmid encoding an array of virulence genes (1). More recently, however, case reports and studies using archived bloodstream isolates have indicated the occurrence, albeit at low frequencies, of hvKp infection in the United States. Since pneumonia is a common clinical presentation of *K. pneumoniae* infection, we sought to investigate whether hvKp is implicated in respiratory tract infection, including pneumonia, and if so, how often.

We prospectively collected *K. pneumoniae* clinical isolates reported from respiratory cultures at four hospitals belonging to a healthcare system in Western Pennsylvania over a 2-year period. These isolates were screened by PCR to detect cardinal virulence genes, which is considered the gold standard in identifying hvKp (8), and also by the string test, which was initially proposed as a screening method for hvKp but has subsequently been associated with low specificity (1). In this investigation of 273 *K*. *pneumoniae* isolates from 216 unique patients, though 11 strains were positive for string test and 4 of them were serum-resistant, only 2 of these strains were identified as genetically confirmed hvKp strains possessing *rmpA*, *rmpA2*, *iutA*, and *iroN*. Of these, only one strain was established as hvKp in a mouse pneumonia model. The overall genetically defined hvKp prevalence of 0.9% that we observed is numerically lower than 3.5%–3.8% reported from recent studies in New York City and Chicago (5, 6). This may be explained by the fact that our study collected respiratory isolates whereas the other studies primarily collected blood isolates or may reflect actual differences in the prevalence of hvKp by locale. Nonetheless, we are intrigued by the fact that the two patients we identified with hvKp were both local residents with active recreational drug use who presented from the community with cardiac arrest. hvKp isolated from these cases likely contributed to aspiration pneumonia that occurred at the time of their presentation, in contrast to the more frequent hvKp presentation of severe community-acquired pneumonia.

Both hvKp strains we isolated possessed pLVPK-like virulence plasmids that were highly similar to pLVPK-like plasmids previously identified from ST23 and ST86 strains (20), indicating that these two strains are likely part of the global spread of hvKp. Given the absence of international links in either case, we posit that these strains may have already colonized the population at a low level in the locale where the study was conducted. In addition to these two strains, there were nine *K. pneumoniae* strains that were string test-positive. These additional strains had virulence scores of 1 or 0, and the only acquired virulence gene detected by Kleborate was *ybt* in two strains, but two of the nine strains demonstrated resistance to normal human serum. We hypothesized that these two serum-resistant, string test-positive strains (2127 and 790) may also display increased virulence in mice, and we opted to use the lung infection model given that the corresponding patients presented with pneumonia. However, *in vivo* studies showed that wild-type mice cleared these strains (790 and 2127) in the lungs and other tissues. This lack of outgrowth from tissues in strains 2127 and 790 may be due to the low inoculum (1–2 × 10^3^ CFU/lung) given to the mice, which was sufficient to induce significant infection with the hvKp strains but may not have been sufficient for non-hvKp strains. This is in contrast to mice infected with strains 1321 and 1665, which had much higher bacterial burdens and were sick at 48 hours post-infection. Furthermore, we observed a significant difference in the survival of mice when infected with strain 1321 compared to strain 1665. Resistance to serum killing, though often used as an *in vitro* screening assay for *K. pneumoniae*, is not an optimal surrogate to predict tissue hypervirulence, and additional studies such as bacterial genetics and a mouse model of infection are still needed to assess for the hypervirulent phenotype (21).

Our investigation has several limitations. The study was conducted at one health system in the Mid-Atlantic United States, and the findings may not be generalizable. We also collected only clinical isolates that grew from respiratory specimens, thus we were not able to compare results with other culture sites. We used *rmpA*, *rmpA2*, *iroN,* and *iutA* as genetic markers to screen for hvKp. Subsequent studies have demonstrated the use of *peg-344*, *iroB*, *iucA*, *rmpA*, and *rmpA2* as having improved diagnostic accuracy in identifying hvKp strains (2). In fact, both the KL1 (1665) and KL2 (1321) strains carried intact *peg-344* on their hvKp-associated virulence plasmids. Finally, we conducted mouse studies, the experimental gold standard for identifying hvKp, on only 4 of 11 string test-positive strains that were serum-resistant.

In conclusion, we identified genetically defined hvKp strains at a low frequency in respiratory specimens of patients without known travel history who presented to a hospital system in Western Pennsylvania, suggesting local acquisition of this pathogen. Furthermore, additional string test-positive, serum-resistant *K. pneumoniae* strains were identified. This work adds to the growing body of evidence suggesting that hvKp, once considered pathogens endemic to Asia, may now be circulating in North America.

## Data Availability

Raw sequence reads and draft genome assemblies have been deposited in the NCBI database under BioProject PRJNA984710, under accession numbers SAMN35995158-SAMN35995168.

## References

[1] Russo T.A, Marr CM. 2019. Hypervirulent Klebsiella pneumoniae. Clin Microbiol Rev 32:e00001-19. doi:10.1128/CMR.00001-19 31092506 PMC6589860

[2] Russo TA, Olson R, Fang C-T, Stoesser N, Miller M, MacDonald U, Hutson A, Barker JH, La Hoz RM, Johnson JR. 2018. Identification of biomarkers for differentiation of hypervirulent Klebsiella pneumoniae from classical K. pneumoniae. J Clin Microbiol 56:e00776-18. doi:10.1128/JCM.00776-18 29925642 PMC6113484

[3] Karlsson M, Stanton RA, Ansari U, McAllister G, Chan MY, Sula E, Grass JE, Duffy N, Anacker ML, Witwer ML, Rasheed JK, Elkins CA, Halpin AL. 2019. Identification of a carbapenemase-producing hypervirulent Klebsiella pneumoniae isolate in the United States. Antimicrob Agents Chemother 63:e00519-19. doi:10.1128/AAC.00519-19 31061159 PMC6591612

[4] Kamau E, Allyn PR, Beaird OE, Ward KW, Kwan N, Garner OB, Yang S. 2021. Endogenous endophthalmitis caused by ST66-K2 hypervirulent Klebsiella pneumoniae, United States. Emerg Infect Dis 27:2215–2218. doi:10.3201/eid2708.210234 34287130 PMC8314818

[5] Kochan TJ, Nozick SH, Medernach RL, Cheung BH, Gatesy SWM, Lebrun-Corbin M, Mitra SD, Khalatyan N, Krapp F, Qi C, Ozer EA, Hauser AR. 2022. Genomic surveillance for multidrug-resistant or hypervirulent Klebsiella pneumoniae among United States bloodstream isolates. BMC Infect Dis 22:603. doi:10.1186/s12879-022-07558-1 35799130 PMC9263067

[6] Parrott AM, Shi J, Aaron J, Green DA, Whittier S, Wu F. 2021. Detection of multiple hypervirulent Klebsiella pneumoniae strains in a New York City hospital through screening of virulence genes. Clin Microbiol Infect 27:583–589. doi:10.1016/j.cmi.2020.05.012 32461145

[7] Ballaben AS, Galetti R, Ferreira JC, Paziani MH, Kress MR von Z, Garcia D de O, Silva P da, Doi Y, Darini ALC, Andrade LN. 2022. Different virulence genetic context of multidrug-resistant CTX-M- and KPC-producing Klebsiella pneumoniae isolated from cerebrospinal fluid. Diagn Microbiol Infect Dis 104:115784. doi:10.1016/j.diagmicrobio.2022.115784 35994834

[8] Yu F, Lv J, Niu S, Du H, Tang YW, Pitout JDD, Bonomo RA, Kreiswirth BN, Chen L. 2018. Multiplex PCR analysis for rapid detection of Klebsiella pneumoniae carbapenem-resistant (sequence type 258 [ST258] and ST11) and hypervirulent (ST23, ST65, ST86, and ST375) strains. J Clin Microbiol 56:e00731-18. doi:10.1128/JCM.00731-18 29925644 PMC6113471

[9] Clinical and Laboratory Standards Institute. 2022. Performance standards for antimicrobial susceptibility testing. 32nd ed. Berwyn, PA.

[10] Tsui CK-M, Ben Abid F, Al Ismail K, McElheny CL, Al Maslamani M, Omrani AS, Doi Y. 2023. Genomic epidemiology of carbapenem-resistant Klebsiella in Qatar: emergence and dissemination of hypervirulent Klebsiella pneumoniae sequence type 383 strains. Antimicrob Agents Chemother 67:e0003023. doi:10.1128/aac.00030-23 37310284 PMC10353355

[11] Wick RR, Judd LM, Gorrie CL, Holt KE. 2017. Unicycler: resolving bacterial genome assemblies from short and long sequencing reads. PLoS Comput Biol 13:e1005595. doi:10.1371/journal.pcbi.1005595 28594827 PMC5481147

[12] Aziz RK, Bartels D, Best AA, DeJongh M, Disz T, Edwards RA, Formsma K, Gerdes S, Glass EM, Kubal M, et al.. 2008. The RAST server: rapid annotations using subsystems technology. BMC Genomics 9:75. doi:10.1186/1471-2164-9-75 18261238 PMC2265698

[13] Lam MMC, Wick RR, Watts SC, Cerdeira LT, Wyres KL, Holt KE. 2021. A genomic surveillance framework and genotyping tool for Klebsiella pneumoniae and its related species complex. Nat Commun 12:4188. doi:10.1038/s41467-021-24448-3 34234121 PMC8263825

[14] Grant JR, Enns E, Marinier E, Mandal A, Herman EK, Chen C, Graham M, Van Domselaar G, Stothard P. 2023. Proksee: in-depth characterization and visualization of bacterial genomes. Nucleic Acids Res 51:W484–W492. doi:10.1093/nar/gkad326 37140037 PMC10320063

[15] Schwengers O, Jelonek L, Dieckmann MA, Beyvers S, Blom J, Goesmann A. 2021. Bakta: rapid and standardized annotation of bacterial genomes via alignment-free sequence identification. Microb Genom 7:000685. doi:10.1099/mgen.0.000685 34739369 PMC8743544

[16] Iwanaga N, Sandquist I, Wanek A, McCombs J, Song K, Kolls JK. 2020. Host immunology and rational immunotherapy for carbapenem-resistant Klebsiella pneumoniae infection. JCI Insight 5:e135591. doi:10.1172/jci.insight.135591 32213713 PMC7205435

[17] Vubil D, Figueiredo R, Reis T, Canha C, Boaventura L, DA Silva GJ. 2017. Outbreak of KPC-3-producing ST15 and ST348 Klebsiella pneumoniae in a Portuguese hospital. Epidemiol Infect 145:595–599. doi:10.1017/S0950268816002442 27788691 PMC9507641

[18] Wyres KL, Hawkey J, Hetland MAK, Fostervold A, Wick RR, Judd LM, Hamidian M, Howden BP, Löhr IH, Holt KE. 2019. Emergence and rapid global dissemination of CTX-M-15-associated Klebsiella pneumoniae strain ST307. J Antimicrob Chemother 74:577–581. doi:10.1093/jac/dky492 30517666 PMC6376852

[19] Machuca J, López-Cerero L, Fernández-Cuenca F, Gracia-Ahufinger I, Ruiz-Carrascoso G, Rodríguez-López F, Pascual Á. 2016. Characterization of an outbreak due to CTX-M-15-producing Klebsiella pneumoniae lacking the bla_OXA-48_ gene belonging to clone ST405 in a neonatal unit in southern Spain. J Antimicrob Chemother 71:2353–2355. doi:10.1093/jac/dkw137 27118773

[20] DeLeo FR, Porter AR, Kobayashi SD, Freedman B, Hao M, Jiang J, Lin YT, Kreiswirth BN, Chen L. 2023. Interaction of multidrug-resistant hypervirulent Klebsiella pneumoniae with components of human innate host defense. MBio 14:e0194923. doi:10.1128/mbio.01949-23 37671860 PMC10653787

[21] Bain W, Ahn B, Peñaloza HF, McElheny CL, Tolman N, van der Geest R, Gonzalez-Ferrer S, Chen N, An X, Hosuru R, et al.. 2024. In vivo evolution of a Klebsiella pneumoniae capsule defect with wcaJ mutation promotes complement-mediated opsono-phagocytosis during recurrent infection. J Infect Dis 230:209–220. doi:10.1093/infdis/jiae003 39052750 PMC11272070

